# Exploring the Possible: A Unifying Cognitive and Evolutionary Approach to Art

**DOI:** 10.3389/fpsyg.2021.787789

**Published:** 2022-03-25

**Authors:** Francis F. Steen, Santanu Chakraborty

**Affiliations:** ^1^Department of Communication, University of California, Los Angeles, Los Angeles, CA, United States; ^2^Department of History of Art, Visva Bharati University, Santiniketan, India; ^3^Program in Life Sciences, Atria University, Bengaluru, India

**Keywords:** artistic exploration, possibility space, perceptual affordances, decision theory, prospective cost, probabilistic value assessment, communicative potential, actualizabiity function

## Abstract

The subjective delight associated with the creative arts poses a well-known challenge to an integrated causal analysis of human psychology. Here we examine the distal causes of art in terms of an irreducibly risky search in a vast phase space of cognition and behavior. To explore means to engage in an activity that may result in a zero or negative payoff. Moreover, you may be unable to assess the risks with any certainty; the costs might spiral out of control. At the same time, the known alternatives may simply not be viable; natural selection has no problems acting on the failure to locate new habitable subspaces. This represents the hard problem of evolution: there is no recurring procedure that will reliably deliver the benefits of a successful exploration. We propose to locate the emergence of play and art in the tension between the irreducible risks of exploration and its potential benefits and examine the complex suite of adaptations that has emerged to solve, however, imperfectly, the hard problem of evolution. This includes adaptations for lowering the cost of exploration and strategies for open-ended yet loosely targeted searches. We argue that the ability to become aware of possible actions, to evaluate their respective merits, and to explore and develop new strategies of perception, thinking, and action have had a major impact on human survival and reproduction and have been subject to persistent natural selection. The arts, we suggest, represent a distinct cognitive mode of pushing the boundaries of what is familiar and known into new areas of perceptual, emotional, and agentive exploration and discovery, characterized by a proximal motivation of intrinsic enjoyment.

## Introduction

Art is experienced as intrinsically interesting and enjoyable, for reasons that are not obvious. Dissanayake (2019) usefully clusters existing evolutionary theories of art into ten different approaches. She likens this diversity of approaches to the story of the blind men and the elephant, in which each person is factually correct, but makes a mistake in the scope of validity of their explanation, illustrating the Jain doctrine of *anekantavada* or many-sidedness. The relatively new field of evolutionary approaches to the arts, she concludes, “awaits a unifying set of principles about its subject.” Dissanayake herself proposes that artification, the act of making art, can be characterized as an act of “making special,” emphasizing how art is perceived to be uniquely valuable. Is it possible, we ask, to characterize the fundamental adaptive problem that art solves in a manner that is compatible with the high value assigned to it?

In the following, we examine the distal causes of art in the occasional and uncertain rewards of an open-ended exploration of the possible. Art and playful behavior, we propose, are activities whose biological function is to allocate surplus resources to solving the hard problem of evolution: how to discover unknown unknowns. We argue that the ability to become aware of possible actions, to evaluate their respective merits, and to explore and develop new strategies of perception, thinking, and action have had a major impact on human survival and reproduction and have been subject to persistent natural selection. The arts, we suggest, represent a distinct cognitive mode of pushing the boundaries of what is familiar and known into new areas of perceptual, emotional, and agentive exploration and discovery, characterized by a proximal motivation of intrinsic enjoyment.

In this way we build on the fifth approach in Dissanayake’s typology, grounding art in adaptations for play (Steen and Owens, 2001; Tooby and Cosmides, 2001; Steen, 2006). To clarify the radical adaptive challenges involved in search, we begin the essay by establishing an explanatory framework of reality as manifest with ontologically grounded but latent possibilities, where the biological function of information is to bring the possible selectively and painstakingly into being. We distinguish between genetic information on the one hand and sensory or cognitive information on the other, arguing that they differ radically in origin but similarly function to enable the organism to intervene in the coming into being of the possible and achieve biologically optimal outcomes. Sensory information, we suggest, alters the adaptive landscape, creating opportunities for new behaviors. In animals, statistical outliers become significant adaptive targets; cognition evolves to access resources unattainable directly by natural selection. Cognition in turn is confronted with infinitely large possibility spaces, most of whose contents is useless or harmful.

It is in the hard problem of locating new modes of meaning-making within infinitely large possibility spaces that we propose to locate the human adaptations for play and art. The full repertoire of actualizability functions invariably captures only a small subset of what is possible; most of the possibility space remains at any given moment unknown and invisible. Innovation requires exploration, which is potentially risky; our exploitation of the possible is guided by existing knowledge and is generally conservative, aiming to avoid dangerous mistakes and expensive failures. Innovation, however, comes with very significant benefits of survival and reproduction. Art and play, we suggest, evolved to solve the problem of how to discover useful strategies in infinitely large possibility spaces in ways that are safe and cheap.

## The Actualizability Function

To prepare the ground for a new and unifying understanding of the biological function of art, we propose to begin by broadening our conception of reality by assigning an ontological status to the possible. It may seem reasonable to restrict the category of being to what is physically present, measurable, a fact. The possible in contrast can be characterized in purely epistemic terms, as something generated by the imagination but devoid of actuality or being. Whatever is not a fact must surely be a non-fact; there is no need for an intermediate category. However, we suggest this fails to capture the essential nature of the possible as a critical dimension of how manifest reality comes to be. The way we conceptualize the relation between the possible and the real, we propose, broadly determines how we understand the biological function of art.

In classical physics, it seemed for a while that the messiness of the possible could be conclusively excluded in favor the singular and predictable order of the necessary. In Laplace’s (1902/1814) famous formulation, a complete description of the position and momentum of every particle would give us the ability not only to predict the future, but to retrodict the past, stretching before and after in a single unwavering chain of causality. This vision of a mechanical universe, however, suffers from an embarrassingly obvious flaw: the laws of physics are not simply descriptions of facts; we use them to act and achieve our purposes. By sending a man to the moon we not only validate Newton’s laws; we also demonstrate that we have the ability to intervene in the causal chain of reality, disrupting its seamless unfolding. How do we explain our ability to carefully and skillfully bring a very specific possibility into being?

The most cogent treatment of the actuality of the possible we find in the discussions surrounding the experimental findings of quantum physics. “In the experiments about atomic events we have to do with things and facts, with phenomena that are just as real as any phenomena in daily life,” Heisenberg (1958) writes. “But the atoms or the elementary particles themselves are not as real; they form a world of potentialities or possibilities rather than one of things or facts” (128). According to the generally accepted Copenhagen Interpretation developed by Heisenberg and Niels Bohr, the trajectory of a particle occupies a superposition of states, each of which has a certain probability of being actualized. Schrödinger formalized this description in the quantum wave equation: in any given situation, the equation has multiple valid solutions, each associated with a particular probability. An act of measurement will invariably find the particle on one of these trajectories; until that act, the system exists in a state of superposition. As Heisenberg puts it, the possible occupies an intermediate state of being. What is possible is not a manifest and actual fact, but facts emerge from the possible in an orderly manner. The quantum physicist Bohm (2005/1980) describes this process as a movement of unfolding from an implicate to an explicate order.

Analogously, we propose, in the act of prediction, cognitive processes formulate an actualizability function. Similarly to the quantum wave function, the actualizability function in any given situation has multiple valid solutions, each associated with a certain probability. Like Schrödinger’s wave function, the actualizability function is a description of a superposition of possible states that have the potential to collapse into a singular reality. Most of these possibilities will typically be unknown; a cognitively realized actualizability function is invariably partial, capturing only a small subset of what is possible. Where the quantum wave function, however, is aimless and collapses in a manner that cannot be controlled, the cognitive actualizability function has preferred outcomes and levers – what quantum theory terms “hidden variables” – to increase the probability of a particular outcome. By identifying and modifying these variables, we engage with the possible as agents, deliberately and carefully seeking to bring our preferred selection of the possible into being. Like the quantum wave function, the cognitive actualizability function is probabilistic and doesn’t always succeed. Our successes may be praised by our supporters as demonstrations of competent expertise and characterized by our detractors as luck; both have a point. Conversely, our partial functions may fail to sufficiently control the necessary variables, leading to outcomes we term “mistakes,” “failures,” “accidents,” and “disasters.”

Natural selection also engages with the process of bringing the possible into being, but in a distinctive manner. On the one hand, genetic information functions to guide the organism through the phase space of the possible and orchestrate the selective actualization of biologically optimal outcomes. On the other, natural selection acts on outcomes and does not make predictions. Genetic information, according to Darwinian evolutionary theory, is formed through random variation, phenotypic expression, and differential survival in ways that at no stage involves an actualizability function. Perception and cognition, in contrast, can be optimized by formulating partial local actualizability functions.

Following the neo-Darwinian synthesis, the variation in form on which natural selection acts is attributed to random mutations in the DNA, caused by processes unrelated to the control of the organism. The assembly of genetic information is “blind” in the sense that it sees neither the real nor the possible. In this model, genes do not collect information of any kind about the world. There are no sensory systems that supply them with information, no causal links or pathways between the domain in which the genes are expressed and the information they contain. There is no process of prediction or anticipation, design, or planning: no inferences are made about the possible based on past and present facts. Instead, the information contained in the genes of living beings is modified only by chance, by the action of external forces – by cosmic rays, nuclear radiation, chemical teratogens – or by accidental errors within the genetic material’s orderly replication and maintenance. The processes that change the information contained in the genes are either statistical or template matching in nature, have no direct relation to the domains in which this information serves a function in the life of the organism. Genetic information has no referent; it is not “about” anything. The neo-Darwinian synthesis asserts that the work of the genes is accomplished without the formation of an actualizability function – there is no superposition of possibilities, no collapse of alternatives into a single reality (in the present context, we adopt no position on whether Darwin was substantively correct in thinking that nature does not directly access the possible; for a challenge, see for instance Payne and Wagner, 2019; Monroe et al., 2022). Nonetheless, genetic information acts exactly like any other form of information: its biological function is to selectively realize highly specific possibilities latent in the local environment conducive to survival and reproduction.

We seek to explicitly highlight these fundamental characteristics of Darwinian evolutionary theory in order to bring out their contrast with cognition. By cognition, we mean precisely the processes that Darwin and Spencer sought to evacuate from their account of natural selection. The biological function of cognition is to accomplish exactly what natural selection is presumed not to be doing: to gather local information, to select and refine it, to infer the possible, to anticipate, to plan. How does this capacity itself evolve? What is the problem it solves?

To appreciate the constructive interplay of sensory and genetic information, consider a free-floating organism that lacks the ability to detect the proteins it needs to ingest. It moves through the water feeding by indiscriminate filtering. Some of its energy is being wasted by feeding when no suitable nutrients are available; this sets up an adaptive potential, a structured possibility space. Imagine a random mutation that gives a neuron on the surface of this organism’s tiny brain the capacity to detect a protein molecule in the water. What matters for the organism is the potential that this information reveals: the difference between the presence of proteins and the absence of proteins is actionable. The organism is capable of feeding under both conditions, but the likelihood that feeding will be productive when the detector is not firing is lower than when the detector is firing. A simple action realizes this potential: by initiating feeding when the neuron fires, it ingests the proteins. It can now filter-feed when they are present and not waste the effort when they are not. This adaptation in turn prepares the stage for a second adaptive potential: a mutation that constructs a second neuron, connected to the first, with the ability to detect the frequency with which the first neuron detects the presence of a protein molecule. The difference in timing between the firings creates a new actionable potential: the organism can now filter-feed only when the firing rate exceeds a certain threshold. Yet this novelty in turn sets up a fresh adaptive potential: a third neuron connected to the second that detects the changes in frequency over time. With this ability, the organism acquires information about differences in its immediate environment that it can utilize to guide its movements: when the interval between detections is dropping, it moves forward; when it increases, it changes direction. The combination of spatial and temporal differences allows the organism to realize a potential for more efficient feeding that had been latent in its environment all along, but that it had been incapable of accessing. This allows us to generalize: the biological significance of information is that it allows the organism to be guided toward actualizing certain possibilities that are latent in its environment.

In this scenario, the organism utilizes information from two very different sources. First of all it collects information from its environment: the difference between the presence of a protein and its absence, the differences in firing frequency over time (speed), the differences in firing speed (acceleration). This sensory information has a referent – the protein molecules in the water at different times and in different spatial concentrations – and is continuously collected by the organism’s sensory neuron.

Secondly, the organism is also dependent on a second source of information: the genetic information utilized to construct the sensory neuron itself, as well as the neural adaptations needed to process and actuate this information. Like sensory information, the biological function of genetic information is to selectively actualize a potential hidden in the environment. The information contained in the genes acts in a vast space of possibilities. Under the right conditions, the DNA assembles organic forms out of molecular building blocks. It selects and orients individual molecules into three-dimensional structures. In the phase space of molecular combinations, it realizes a unique set of permutations: specific and unlikely sequences of precisely oriented molecules. Its work can be visualized as a complex act of navigation in a multidimensional phase space. Through this act of navigation, genetic information manifests a potential hidden in its immediate local environment, a vanishingly improbable, complex and precisely determined biological structure that furthers its survival and reproduction. Yet this information was never collected by the organism. In fact, according to Darwinian evolutionary theory, the information that was utilized to construct the neuronal sensor was never collected at all, does not have a referent, and does not express a difference between anything.

The evolution of sensory organs producing sensory information, however, alters the possibility space of natural selection. Once information about the presence or absence of proteins in the water is available to the organism, there is an adaptive potential to utilize it. Natural selection doesn’t see this possibility; causally unrelated processes must intervene by chance to generate the relevant change in the genome to construct the appropriate neurological adaptations that allow the organism to act on the new information. The randomness and consequent temporal demands of this process, we suggest, creates a powerful adaptive attractor for the evolution of the capacity to formulate even a minimal actualizability function. As increasingly rich sensory information reshapes the adaptive landscape, an organism that by chance evolves this capacity will have a persistent competitive advantage in a wide range of circumstances.

Once a neuron is capable of collecting information about the presence of protein molecules in the water, an adaptive potential is created in which the organism would benefit from utilizing this information and also from refining it over time. In our hypothetical organism, the factual information captured by the sensory neurons opens up specific possibilities: to ingest proteins when feeding, to set a threshold for a minimal meal, to navigate toward higher concentrations. Each of these adaptations have a fat middle: natural selection will eventually be able to locate the appropriate solution. Yet as the wealth and complexity of sensory information increases, a dense and complex thicket of fleeting adaptive potentials will arise that the random process of natural variation and selection would be too slow to efficiently exploit. Tooby and Cosmides (2000) develop a similar distinction: natural selection generates adaptations for handling scope operators, but it cannot determine what their local values should be.

Statistics provides a useful perspective on this dilemma that natural selection cannot solve directly. Because of its random nature and slow rate of innovation, natural selection can only exploit patterns that persist over time – the fat middle of the distribution of events. Sensory information, however, in certain circumstances creates adaptive landscapes where the main opportunities are located in the outliers. Once an organism is motile, the adaptive landscape fills with momentary possibilities that natural selection is too slow to exploit. In the fossil record, what emerges with motility is cognition: natural selection favors the investment in sufficient neural structure – a notochord, a brain – to support the systematic exploitation of statistical outliers. Evading a predator or catching a prey represent adaptive opportunities with such a dramatic payoff that a complex neurological infrastructure becomes a justified and sustainable strategy for exploiting them. Natural selection, by random variation and carefully controlled phenotypic outcomes, has created cognitive systems capable of exploiting fleeting statistical outliers.

What these cognitive systems do is to gather information about the facts and use that information to formulate actualizability functions: to make predictions about what could happen next. These predictions take the form not only of hypotheses about what is likely to happen, but importantly also develop potential strategies for intervening in the actualization of the possible to achieve a biologically optimal outcome. Each moment in time and space is associated with a particular horizon of possibilities, or action affordances (Gibson, 1977). Each action affordance in turn is associated with an estimated, prospective cost and benefit. In predation, the potential cost to the prey is infinite, but it is still vital to minimize the cost of evasion; in foraging, the energy acquired must on average exceed the energy expended. To formulate a useful actualizability function, the challenge is to identify a subset of the possible where prospective benefits are maximized and prospective costs minimized.

In short, natural selection is far too slow to be able to effectively exploit the rapidly changing adaptive landscape in the life of an animal. In the evolution of cognition, natural selection builds information processing structures that have the ability to identify and take advantage of individual opportunities, rare statistical outliers, that are inaccessible to natural selection. Cognition accomplishes this by prospectively accessing the possible, the latently real; by forming probabilistically structured actualizability functions, partial and cognitively expensive; and by deliberately intervening in the orderly actualization of the possible into the real. Natural selection will favor the elaboration of such cognitive systems in rapidly changing adaptive landscapes where survival and reproduction is promoted by ceaseless innovation. Cognition is an adaptation that accomplishes what is beyond the reach of natural selection.

Living organisms in general utilize information to optimize their survival. A hare seeing a fox makes predictions about what the fox is likely to do and takes evasive action. That prediction accesses the possible: one set of events that would lead to being eaten, another set that would lead to a successful escape. Cognition engages with the possible as a latent reality, gathers and synthesizes precise predictive information, and guides behavior to ensure survival even when this is a highly improbably outcome. Mining the possible is hard, but the rewards can be considerable. A recent study concludes it was the smartest avian dinosaurs that survived the Cretaceous-Paleogene extinction event (Torres et al., 2021). Human brain size peaked around the time of the last glacial maximum (Henneberg, 1988), a time of extremely challenging conditions for survival.

Human lives play out within vast fields of latent possibilities: actions you could take, thoughts you could have, skills you could learn, decisions you could make, goals you could achieve. We live in a world that ceaselessly forks and we actively participate in this seething multiplicity of possible futures. It is important to acknowledge that this participation incurs an inescapable cost and is far from free – in this sense, the notion of “free will” is a hangover from a dualistic conception of mind and matter; what we have is “costly will.” Our ability to participate in the unfolding of the possible into manifest reality persists in spite of continuing attempts to universalize determinism in neuroscience (see for instance Roskies, 2006) and physics (see for instance Hossenfelder and Palmer, 2020). In our personal lives and relations, in politics and in the courts of law, it is in practice universally accepted that we as agents possess the ability to manifest specific aspects of a latent space of possibilities, that we struggle to identify and realize the best possible version, and that our hopes and fears are responses to something that is true, something that is actualizable, even if it is not yet manifest and real. Truth, in this sense, overflows the real and embraces the possible. It may be true that we will meet in Rome in May, it may be true that you can learn to play the violin. These are not facts, they are not empirical data points, yet such latent possibilities are precisely what guide and unfold human action in costly and orderly ways.

Actualizability functions are flexible assessments designed to generate one-off predictions and identify useful outliers. The information assembled in an actualizability function represents a tiny and incomplete subset of ways in which the possible may unfold into the real. Each cognitively realized function is partial, serving to identify a few promising possibilities and a few of the spatiotemporal actions that may change the course of events. It specifies that a finite and feasible amount of effort will with some degree of probability nudge one out of a superposition of multiple possibilities into being. Yet these predictions are not fantasies; they do not belong to the class of fictions. Our assessments of how the possible can be brought into being are grounded in networks of statistical knowledge gathered by experience. Their subject matter is not primarily established facts but the probabilisitic processes whereby rich superpositions of possibilities can be selectively nudged to emerge into reality.

The world, we suggest, is not only subject to uncertainty due to imperfect knowledge – what we might term epistemic indeterminacy. More fundamentally, it is subject to a radical ontological indeterminacy. In any given moment, the world is underdetermined and unfinished, emerging out of a vast superposition of actual possibilities. Life is enabled by this ontological indeterminacy; natural selection builds the informational structures that allows life to exploit persistent statistical patterns in the possible for biologically optimal outcomes and turn them into realities. Cognition solves the even harder problem of exploiting statistical outliers, rare opportunities affected by multiple variables, even non-recurring one-offs. It is in the crack between what could be and what is that our lives emerge, not simply happening, but deliberately brought into being.

In this section, we have argued that the biological function of information is to selectively realize the possible. This is true of sensory information, which the organism utilizes to guide its behavior in space and time. It is similarly the case for genetic information, which assembles precise organic forms out of vast phase spaces of different molecules. Life must navigate a complex field of possibilities and choices which are infinite in character. Survival is a matter of having adequate information to be able to selectively nudge biologically optimal possibilities into existence. Through their motility, animals are exposed to rapidly changing adaptive landscapes, fleeting possibilities that statistically speaking are outliers, non-recurring opportunities that natural selection cannot directly exploit. The evolution of cognition is enabled by this particular adaptive landscape. A central innovation of cognition is the actualizability function, or the ability to model multiple a suite of superposed possible trajectories through a cascading succession of affordance horizons. Actualizability functions include a representation of the likely costs and prospective benefits of a particular strategy; these assessments are informed guesses subject to continuous updates and adjustments in an ongoing process of optimization.

## Art and the Possible

Art can be recruited for any number of purposes, such as impressing potential mates or seeking to enhance one’s social status. Yet the problem that art evolved to solve is far more fundamental: to explore the infinitely large human possibility space for new and interesting forms of order. The study of artistic expressions provide a unique window into the deep structure of the challenge of survival and reproduction, suggesting that complex patterns or orders, chains of similar differences and different similarities (Bohm, 2004) play a vital role in formulating the complex actualizability functions that serve to bring the tiny biologically favorable subset of the possible selectively into being.

To explore the possible for fruitful actualizability functions is inevitably hard. The fields with which cognition engages are infinite in multiple dimensions. Each component skill an organism possesses increases factorially the size of its phase space of action: if you have ten skills, they can be combined into 3,628,800 non-repeating action sequences. A deck of cards can form 52! sequences, a number larger than the number of seconds since the Big Bang. Ballpark estimates place the number of possible chess games around 10^120^; the number of atoms in the observable universe is thought to be between 10^78^ and 10^82^. A brief window of a human life is far more complex than chess, with an infinitude of latent variables and possible moves. As we engage with our environment, each skill creates a complex and rapidly changing horizon of affordances, dynamically multiplying without bound the number of possible action sequences extending into an increasingly uncertain future. Moreover, the vast majority of what is possible is useless or downright harmful; venturing out into the possibility space of human actions and human relations in an exploratory manner can quickly have irreversibly damaging consequences.

We need to sustain our lives by continually realizing outcomes that are statistically wildly improbable, yet the exploration of the infinite spaces in which our lives precariously persist as well as confidently thrive must be carried out in finite time with finite resources. The outcome is necessarily uncertain: an actualizability function is a hypothesis, consisting of a brief or extended cluster of alternative paths forward, each associated with a prospective cost in the form of an investment of time, attention, and effort, as well as a prospective benefit in the form of a future realized state. Since these costs and benefits are prospective, their assessed likelihood of being actualizable at the proposed effort is uncertain and require dynamic updating. The potential computational costs quickly spiral out of control, in practice limiting the visibility of the horizon. The opportunities at stake are too fleeting for natural selection to locate; instead, cognition evolves as a control system designed to optimize prospective benefits relative to prospective costs. The task of optimization has no once-and-for-all solutions; it must instead look for effective heuristics to find good-enough solutions in finite time.

The infinite size of the possibility spaces we engage with combined with our imperfect understanding of them and our finite resources available to explore them create a continuum of tradeoffs between exploiting the familiar versus exploring the unfamiliar (Mehlhorn et al., 2015). On all scales of life, from the immediate decision to the long-range plan, the prospective costs and benefits of the exploration of new possibilities are pitted against the more controlled exploitation of resources you are relatively confident you can access. The question of how to optimally allocate resources to exploration rather than to exploitation is widely acknowledged to be one of the more fundamental challenges in evolutionary theory and decision-making (Cohen et al., 2007; Gopnik, 2020; René Traoré et al., 2021; for a historical overview, see Almahendra and Ambos, 2015). The difficulty of the multi-choice field problem has been studied in the finite case and is known as the multi-armed bandit problem in optimization theory. We note that even the finite case, choosing the optimal strategy where n different choices exist each with an unknown probability of success, was considered intractable until the later half of the twentieth century (Gittins, 1979). Inspiration for local solutions to certain classes of human problems have been found in foraging behaviors across a wide spectrum of animals, from the adaptive tuning of the exploitation-exploration trade-off in four honey bee species (Young et al., 2021) to the strategic decision-making observed during the bubble-net hunting behavior of humpback whales (Mirjalili and Lewis, 2016; Wu et al., 2019). In real-life situations, genetic as well as cognitive information is invariably incomplete, precluding stable solutions to the exploration-exploitation tradeoff dilemma.

It is in this tradeoff that art emerges as a suite of adaptations for decreasing the cost of exploration and increasing the likelihood of discovering something of value. Faced with infinitely large, mostly unknown, and potentially wasteful or downright dangerous possibility spaces, organisms will be strongly encouraged by natural selection to play it safe and stick to what works. Any stability in the strategies of one organism, however, will create new opportunities for other organisms to exploit it. Natural selection will therefore strongly favor innovation. When the stability of exploitable strategies drops below the time required for reproduction, natural selection responds by evolving cognitive control systems.

The origins of art can be traced back to a suite of adaptions in this control system for lowering the effective cost of exploration and raising the likelihood of finding something of value. First, Burghardt’s (2005, 2014) key finding is that animal play typically takes place under conditions of metabolic surplus. By utilizing excess resources in exploration, we lower the opportunity costs. Gopnik (2020) argues for a life-history division of labor, where the young, typically still nourished and protected by their parents, privilege exploration and the mature exploitation and presents evidence that preschool children under certain conditions will outperform trained scientists in scientific discovery. What this model doesn’t capture is the undisputed persistence of lifetime creativity, honored in the social category “artist:” just as parents are willing to support their children’s materially unproductive playful and exploratory behavior, societies are typically willing to support a limited number of adults in their creation of art (Brown, 1991).

Second, as Steen and Owens (2001) argue, play involves a distinct self-constructive mode that radically transforms the affordance horizon. In play, a friend can serve in the place of an adversary, a play bite can stand in for a real bite (Bateson, 1972), a table can function as a landing pad for a toy spaceship. The exploration of possibilities in play and art makes heavy use of simulations, shifting canonical affordances to a fluid use of material anchors. Non-human animals engage in cognitively sophisticated forms of object pretense. For instance, a cat may pretend that a ball of yarn is a mouse and treat its movements as if they were attempts by the mouse to get away. In pretending, the cat dramatically expands the material circumstances in which it can practice pouncing and hunting. Through such cheap and safe playful practice, the maturing cat develops new actualizability functions. A child may pretend to construct a house for dinosaurs out of cardboard and a rag doll, constraining the complexity of the task to a pedagogically optimal challenge and radically lowering the risks and material costs. Humans expand on object pretense through agent pretense. In agent pretense, a group of children may pretend to be a family of leopards nesting in a tree. An actor may pretend to be a fictional character. In developing this fictional character, the actor generates an alternative identity, characterized by a different history, a different set of affordances, a interesting set of goals placed in some relation to fictive resources available. Drawing on infinitely large possibility spaces to develop an optimally formulated fictive world, the actors explore patterns of human relations, decision making, and high-level cognitive skills, some of which can be exported into real life. Yet it is an striking feature of the human visual and episodic imagination that it is not constrained by what is plausible; magical capabilities are attractive to explore even in situations where the means to realize them appear absent. This indicates a radically calibrated search function that has the potential power to penetrate into unknown unknowns and generate unprecedented innovation.

Complementing adaptations that serve the biological function of making the exploration more affordable we find adaptations that serve the function of increasing the likelihood that the exploration will be fruitful. These can be characterized as search heuristics; they constitute a rich field of scientific investigation and indeed is where many contemporary evolutionary theories of art can be situated.

An adaptation for increasing the probability that a search will be fruitful we can characterize as exploration bias. Ever since the Paleolithic, artistic depiction has been drawn to the fertile female body, a clear candidate for an evolved exploratory bias. Similarly, studies indicate that people are drawn to representations of landscapes that in our evolutionary history provided promising resources for survival, as argued in evolutionary aesthetics (Voland and Grammer, 2003; Falk and Balling, 2010). Exploratory biases define favored subspaces in the infinite vastness of possible patterns, shapes and forms. The subspaces constitute lesser infinities whose exploration provide a hook into evolved preferences, yet these preferences constrain rather than control the exploration.

Play and art also have strong social dimensions that serve to dramatically lower risk. An inexperienced child faced with an infinitely large and often dangerous possibility space can navigate with reduced risk into this space simply by following more experienced role models, plausibly an evolved behavior. Role models can be identified directly by their superior skills or indirectly through outcomes such as confidence and happiness that correlate with skills. Role models allow us to learn not only to extend current or familiar skills into new areas (known unknowns), but also to be introduced to entirely novel and unexpected skills (unknown unknowns). Children and teenagers may for instance be inspired by their role models to persist through difficulties – a highly generalizable strategy with rich applications (cf. Herrmann et al., 2016). Artistic practices are similarly passed down from generation to generation, forming traditions that consistently show a preference for certain subspaces.

Within a cultural tradition, we can observe that artistic exploration is drawn to aspects of reality that are perceived and experienced as important, whether or not they have persisted long enough to acquire an evolved bias. Over tens of thousands of years, Paleolithic art is primarily focused on the depiction of large animals. However, we interpret these depictions, they inform us of a strong preference for exploring a particular subspace of the possible that is anchored in daily experience. The detailed depictions of the Chauvet horses, for instance, indicate a high level of interest and expertise in the appearance of horses. With the advent of the Neolithic, warfare emerges as a central theme of art. Natural selection has in part handed off decision-making as to the appropriate subdomains for art to explore to the cognitive control system.

Artistic exploration forms a kind of knowledge that is built incrementally, both on an individual and a collective level. Artistic forms of expression favor the exploration of the boundaries of the familiar, generating variations on a theme. Dissanayake (2019) argues that “familiarity with traditional societies makes clear that ‘creativity’ and individual showing off are typically, if not always, discouraged.” Yet this is surely a mischaracterization; traditional societies typically produce art that by any canonical baseline is wildly creative, albeit constrained within subspaces that may persist over long periods, elaborating on familiar and shared themes. Creativity is thus constrained and harnessed, but it is not devalued. Conversely, a theory of art needs to account for the fact that societies may attach value to its ability to destabilize the existing order. By creating a non-conforming work of art, the artist invites the audience to immerse into a different way of being and seeing; the work of art becomes a gateway to new possibilities. In this way art challenges and destabilizes habitual ways of thinking, feeling, and seeing; it can be resisted and rejected; and it can support joint discovery and create new perceptual norms that support communities of meaning. Art opens the mind’s interpretive heuristics and invites the viewer to leap into a new order, a new set of emotions, a new disposition to the world.

In the act of creation, the artist enters a creative state of mind that explores how reality can be constructed. In creating an object of art, such as a dance, a decorative pattern, or a musical composition, the artist brings a new order into being. The creation is tentative; artification moves in a vast phase space of possible choices of materials and arrangements. By engaging directly with the possible, for instance by creating plans for the work of art in the form of imagined concepts or manifest outlines and sketches, it creates a manifest or virtual superposition of states, each of which can be evaluated for its merits. Such drafts and sketches are tentative proposals to the sensory systems; each represents a proposition. In this activity, the artist is engaging directly with the processes that construct perceptual reality on the basis of sensory data. The perceptual systems respond to the material objects the artist creates. Lines of charcoal on a rock wall or shapes of clay create novel and meticulously manipulable perceptual affordances. The artist experiments with how the visual system responds to what he creates in an intimate dialog with the medium. In a play of sensory qualities – of form, movement, pacing, color – artification uncovers new orders.

The discovery of new orders is a foundational cognitive process or activity. The repertoire of orders possessed by the mind circumscribes a combinatorial ability to engage productively with the possibility spaces of reality. Orders of similar differences, as in the steps of a dance, are used to orchestrate our movement through cascading possibility spaces (Bohm and Peat, 1987). Flexible and interrupted rhythms guide our physical movement in time and space. Complex patterns of similar differences and different similarities, of analogies and disanalogies (Fauconnier and Turner, 1998), organize our tasks into flexible alternations of repetition and change that allow us to reach distant and implausible or unlikely goals. In this exploration, the artist may draw attention to the constructed nature of reality as a way to highlight aspects of the human possibility space that typically gets obscured by habit. The process of reality construction involves actualizability functions with multiple solutions, explicitly demonstrated in the phenomenon of bi- and multi-stable percepts (Atmanspacher and Filk, 2013). What these experiments demonstrate is that the simultaneous derivation and contemplation of multiple possible solutions takes place below the threshold of conscious awareness.

In exploring the possibility space of perception, emotion, cognition, and action, the artist pushes against the boundaries of the known. The creation of a work of art can possess a quality of open-ended exploration, a movement into the unknown unknown. Similarly, presented with an opportunity to engage with a work of art, the spectator or audience member goes through a series of stages. First, they must consider whether to allocate resources to the act of appreciation. This involves the creation of a simple actualizability function: they consider different options, create a superposition of opportunities, and select a particular course of action. Secondly, the spectator creates a communicative potential for appreciating the art, an act that goes beyond the actualizability function. In generating an open-ended communicative potential, the spectator is not simple accessing the possible as something that is latently real. Rather, they are accessing the latently possible, that which might lend itself to becoming possible, a gate into the unknown, a tremor in the fabric of reality. Keats (1899/1817) characterized the key quality “to form a Man of Achievement” as a “negative capability:” “when a man is capable of being in uncertainties, mysteries, doubts, without any irritable reaching after fact and reason” (277). The ability to generate a radically uncommitted state may be considered an essential dimension of creativity, taking discovery beyond combinatorics. Conceptually, it parallels Bohm (2005/1980) notion of a superimplicate order out of which the implicate order unfolds.

## Conclusion

In developing an evolutionary or Darwinian account of art, researchers have proposed hypotheses that subsume art within an adaptive territory of familiar biological challenges: to identify promising habitats, to impress the opposite sex, to increase one’s social status. These instrumental hypotheses propose to reduce the biological function of art to a subsidiary strategy for solving known and circumscribed problems that already have other solutions. On the flip side, they fall short of explaining the subjective delight and fascination associated with artistic experiences; they struggle to account for the prevalence of children’s art; and they steamroller over the unique value cultures typically place on artistic behaviors and objects of art. We propose instead that art is a unique adaptive strategy of exploring infinite possibility spaces of perception, motion, and thinking for contextually promising innovations.

In the first section we develop a framework for thinking coherently about the importance of agency within the context of Darwinian evolutionary theory. Life’s ability to survive, we suggest, is grounded in the latent superposition of a multiplicity of states that we commonly call possibilities and opportunities. The possible is not merely an epistemic construction, a matter of incomplete knowledge; it refers to an unmanifest or latent level of reality, an ontological indeterminacy, that can be selective nudged into biologically optimal realizations.

What orchestrates this act of selective realization is information. We distinguish between genetic information, generated by natural selection without a referent, and sensory-cognitive information generated by the senses and cognitive processes. These two different kinds of information mutually interact and alter each other’s possibility spaces. While genetic information is only able to take advantage of stable and recurring features, sensory-cognitive information creates opportunities for exploiting fleeting statistical outliers. Cognitive processes accomplish this by formulating actualizability functions that model the superposition of possibilities, guiding the organism to intervene strategically to selectively unfold the possible into reality in a probabilistically controlled manner.

We summarize our thesis by suggesting that the biological function of art is to develop new actualizability functions: to explore new possible orders and selectively bring them into reality. It achieves this across the spectrum of human perception, emotion, thought, and action. While the average payoff of art across small intervals of time may appear low, across larger intervals innovation is essential.

In the second section, we argue that at any moment in an animal’s life, the horizon of affordances is infinite, meaning the cost of modeling it prospectively in its totality is invariably unaffordable. While the size of the possibility space is astronomically vast, an infinitude of infinities, most of it is either useless or antithetical to survival and reproduction. The strategies that sustain life are rare, hard to find, and statistically unlikely. They do not happen by accident; they happen only when the appropriate information is acting. While this means that the risk of exploration is high, within these vast deserts of uselessness are nuggets of gold, exceptional solutions, transformative adaptations. Especially in changing environments, survival may depend on successful exploration.

Natural selection, we suggest, has built a series of varied adaptations for lowering the risk of exploration. With Burghardt (2005), we argue play is a surplus activity. With Gopnik (2020), we note the evidence for a lifetime division of labor: children are strongly predisposed to exploration; adults strongly predisposed to exploitation. Creative exploration, however, is essential at any moment in life and adults retain the desire and ability to explore. With Steen and Owens (2001), we argue that exploration involves a distinct self-constructive cognitive mode, characterized by a relaxation of the canonical constraints of the perception of affordances, the systematic use and development of material anchors, and the extensive use of simulations.

Art is grounded in adaptations whose biological function is to lower the cost and increasing the likelihood of identifying new and valuable orders, actionable patterns of perception, emotion, and action. Art explores new ways of being in the world that have the potential to be uniquely valuable. Dissanayake (2019) proposes that the core of art is the act of “making special”. Let us turn this around and suggest that the act of making art involves a search for a manifest form that is valuable because of the affordances it provides for perceiving and experiencing order. Making special is the individual or collective recognition that we have searched in infinitely vast spaces, constrained only by heuristics, and discovered something of distal value, a source of proximal pure delight.

## Author Contributions

FS developed the details and wrote the text. FS and SC jointly developed the core ideas, contributed to the article, and approved the submitted version.

## Conflict of Interest

The authors declare that the research was conducted in the absence of any commercial or financial relationships that could be construed as a potential conflict of interest.

## Publisher’s Note

All claims expressed in this article are solely those of the authors and do not necessarily represent those of their affiliated organizations, or those of the publisher, the editors and the reviewers. Any product that may be evaluated in this article, or claim that may be made by its manufacturer, is not guaranteed or endorsed by the publisher.
